# A Novel PAN-GO-SiO_2_ Hybrid Membrane for Separating Oil and Water from Emulsified Mixture

**DOI:** 10.3390/ma12020212

**Published:** 2019-01-10

**Authors:** Noman Naseeb, Abdul Azeem Mohammed, Tahar Laoui, Zafarullah Khan

**Affiliations:** 1 Department of Mechanical Engineering, King Fahd University of Petroleum and Minerals, Dhahran 31261, Saudi Arabia; nomannaseeb@live.com (N.N.); zkhan@kfupm.edu.sa (Z.K.); 2 Department of Mechanical and Nuclear Engineering, University of Sharjah, Sharjah 27272, UAE

**Keywords:** hybrid, emulsion, membrane, hydrophilicity, antifouling

## Abstract

In this article, we report the development of a polyacrylonitrile-graphene oxide-silicon dioxide (PAN-GO-SiO_2_) hybrid membrane for separation of oil and water from their emulsified mixture. The membrane was successfully fabricated using a one-step electrospinning process. GO and SiO_2_ nanofillers were added in PAN in different concentrations to determine the optimized composition for the PAN-GO-SiO_2_ hybrid membrane. A scanning electron microscopy (SEM) examination showed that the nanofillers were uniformly embedded in the nanofibrous structure of the electrospun hybrid membrane. The GO was mainly embedded inside the PAN nanofibers, causing knots while SiO_2_ nanoparticles were found embedded on the nanofiber surface, resulting in the formation of micro-nano protrusions on the fiber surface. The formation of these hierarchical structures, together with enhanced hydrophilicity due to oxygen containing groups on both SiO_2_ and GO, resulted in a high rejection (>99%) of oil from oil-water emulsion. Membrane performance evaluation under gravity separation tests showed that the separation flux and phase rejection was enhanced with the incorporation of nanofillers. The inclusion of nanofillers also enhanced the mechanical properties of the membrane. The best flux and phase separation performance was obtained for an optimized concentration of 7.5 wt % SiO_2_ and 1.5 wt % GO in PAN. The flux of separated water was enhanced from 2600 L m^−2^ h^−1^ for pristine PAN to 3151 L m^−2^ h^−1^ for PAN-GO-SiO_2_. The hybrid membrane also showed good mechanical and chemical stability, and antifouling propensity.

## 1. Introduction

Many industries such as oil and gas, textile, food, chemical, and metal processing are generating enormous amounts of oil and water mixture. This contaminated mixture is often discharged in water bodies, causing serious environmental problems [1,2]. The oil contaminations can be broadly classified into free, dispersed, and emulsified oil, with the main difference being the size of oil droplets. Among all, emulsions with a droplet size of less than 20 microns are the most stabilized and difficult to separate oil-water mixture [3,4]. The use of conventional techniques such as gravity settling, coagulation, flotation, flocculation, ozonation, and chemical methods are found ineffective for treating such oily water mixtures due to high operational cost and the problem of getting secondary pollutants as by-products [5,6,7]. Among many other oil-water separation techniques, membrane base separation technology is becoming much more versatile due to its simplicity, high operational efficiency, and environmental friendliness [8,9]. Different methods such as phase inversion [10,11,12], coating [13,14,15], sol-gel [16], and electrospinning [17,18,19,20,21] are being used to produce these membranes. Among all methods, electrospinning is a simple and efficient process used to produce highly porous polymer-based membranes with desirable surface chemistry and high permeation rate [22,23]. Two types of membranes are generally being considered for oil-water separation: hydrophobic-oleophilic and hydrophilic-oleophobic. Hydrophobic-oleophilic membranes will allow oil to pass through the membranes while rejecting water. In these types of membranes, oil causes pore blockage, which consequently results in degrading the membrane’s performance and effective reusability. Reusability limitations of oil-water separation membranes is a major concern that hinders their more widespread commercial viability. Additionally, the available membranes are found to turn brittle when exposed to oily water, thus limiting multiple usage. To solve the issue of fouling, the hydrophilic-oleophobic variant is receiving much more attention from researchers where high hydrophilicity together with hierarchical surface morphology causes water to pass through the membrane, providing a high water flux with oil being repelled over the fiber surface [24,25,26]. 

In the present work, we report the development of electrospun nanofiber membranes, which allow their repeated usage for separating oil and water from their emulsified mixture under gravity situation. Pure polyacrylonitrile (PAN) based membranes and PAN membranes blended with nanofillers such as silica and graphene oxide have been developed in this study. PAN is chosen as a starting material due to its intrinsic high hydrophilicity, compatibility with other additives, high chemical stability, proven electrospinability, and ease of availability. Recent studies demonstrate that the work has largely focused on development of either PAN-GO or PAN-SiO_2_ composite electrospun membranes by utilizing multistep processes. To the best of our knowledge, no one has yet explored the effect of simultaneous addition of both of these nanofillers in PAN to develop a hybrid three-component electrospun membrane. Thus, the development of PAN-GO-SiO_2_ hybrid membranes through a simple one-step electrospinning process can be considered a novel undertaking. In this work, nanofillers of GO and SiO_2_ were added in PAN in different concentrations in order to determine the optimized composition for producing a workable PAN-GO-SiO_2_ electrospun nanofiber hybrid membrane.

## 2. Materials and Methods

### 2.1. Materials

PAN with a molecular weight (Mw) of 150,000 g/mol and *N*,*N*-Dimethylformamide (DMF, 99.8%) were purchased from Sigma Aldrich (St. louis, MO, USA) to prepare the electrospinning solution. Silica nanopowder (SiO_2_) with an average particle size of 12 nm was purchased from Sigma Aldrich while graphene oxide (GO) with flake size in the range of 0.3 to 0.7 microns with a carbon/oxygen ratio of 4:1 was purchased from Graphene Supermarket (Calverton, NY, USA). All chemicals were utilized as received without any further treatment. Deionized (DI) water obtained from Millipore Milli-Q water purification system was used for purposes such as rinsing, cleaning, and dilution during this study. Lubricating oil from Shell, and distilled water were used in making the emulsified oil-water emulsion. 

### 2.2. Fabrication of Nanofiber Membranes

**Figure 1:**
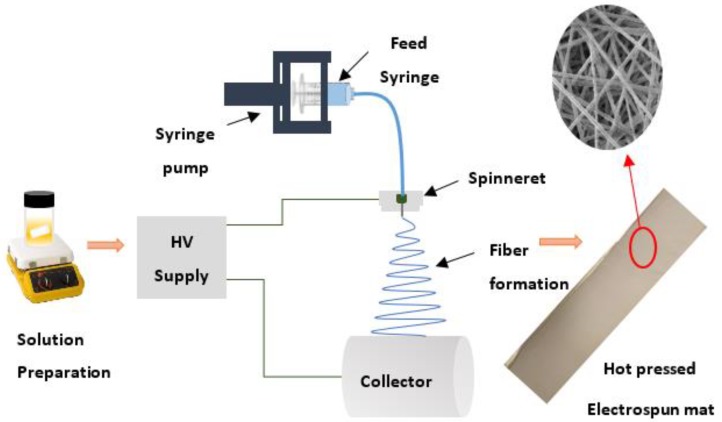
A schematic of electrospinning and PAN based membrane fabrication.

Fabrication of nanofiber membranes was carried out using a Nanon-01A electrospinning setup from MECC CO., LTD (Fukuoka, Japan). PAN, PAN-SiO_2_, PAN-GO, and PAN-GO-SiO_2_ membranes were prepared by first dissolving desired amounts of PAN in DMF through magnetic stirring for 24 h at 60 °C. Silica and/or graphene oxide were then separately dispersed in dimethyl formamide (DMF) with the aid of ultrasonication for 6 h. This dispersion was then mixed in the prepared PAN solution through magnetic stirring for 12 h to obtain a homogeneous solution of PAN-SiO_2_, PAN-GO (for the composite membranes), and PAN-GO- SiO_2_ (for the hybrid membrane). The resultant solution compositions are labeled in Table 1. The doped (stable) solutions containing different ratios of nanofillers were electrospun at an optimized voltage of 20 kV. The other process parameters such as feed rate, spinneret-to-collector distance, collector rotation, spinneret speed, needle size, and temperature were kept constant at 1.0 mL/h, 15 cm, 300 rpm, 70 mm/s, 21 G, and 25 °C respectively. Aluminum foil was wrapped around the drum collector to ease the removal of electrospun mats. A schematic of the procedure is shown in Figure 1. The electrospun mat was removed carefully from the drum collector after the process and dried in a conventional oven at 80 °C for 12 h to completely remove the solvent from the mats. These mats were then hot pressed at 140 °C for 2 min using an electric iron at a hand held pressure of approximately 3 kPa to obtain mechanically robust and dimensionally uniform membranes.

### 2.3. Characterization of Prepared Nanofiber Membranes

The morphology of the electrospun nanofibers was examined using a high resolution, dual beam field emission scanning electron microscope (FESEM) manufactured by LYRA-3, TESCAN, Czech Republic. Prior to examination, the membrane samples were sputtered with gold for 40 s using an ion coater model JFC-1100 from JEOL (Peabody, MA, USA) to mitigate charging associated with non-conductive materials. The surface morphology of individual electrospun nano fiber was studied by collecting them on 150 mesh copper grids and examining them using a JEM-2100F (JEOL, USA) Transmission Electron Microscope (TEM). Fourier Transform Infrared Spectroscopy (FTIR) was used (Nicolet 6700, Thermo scientific, Waltham, MA, USA) to characterize the functional groups in both pristine PAN and PAN based hybrid membranes. An overall spectrum range of 4000–600 cm^−1^ was explored in the transmittance mode. The membranes were analyzed for surface wetting behavior through contact angle measurements under sessile drop method using an optical contact angle goniometer (DM-501, Kyowa interface Science Co. Ltd., Niiza, Saitama, Japan). Water contact angle and underwater oil contact angle was measured for each membrane. The pore size of the fabricated membranes was determined by the expulsion of liquid through gas pressure using capillary flow porometer (3 Gzh, Quantachrome Instruments, Boynton Beach, FL, USA) capable of measuring pore sizes from 500 µm down to 30 nm. Porosity (P %) of the membranes was determined by using the density of the electrospun membrane $\rho_{e}$ and bulk density of the precursor powder $\rho_{p}$ according to Equation (1):

(1) $$P\%=1-\frac{\rho_{e}}{\rho_{p}}\times 100$$

For porosity calculations, the membrane density was evaluated by first determining the total volume of the membrane by dimensional measurements of membrane length, width, and thickness with the aid of a ruler and a low force (0.01 N) digital measuring system (Litematic VL-50, Mitutoyo, Kawasaki, Japan), followed by measuring the weight of the membrane samples. Tensile strength of the membrane samples was determined by using an ElectroForce 3200 test instrument (TA Instruments, New Castle, DE, USA) with a 225 N load cell at a constant strain rate of 0.08 mm/s. The tensile test samples were cut with a die in accordance with ASTM D 1822.

### 2.4. Oil-Water Separation Test

To evaluate the oil and water separation behavior of the fabricated membranes, an oil-in-water emulsion containing 10% lubricating oil was prepared using 2000 ppm lubricating oil in deionized water using high speed stirring at 7000 rpm for 1 h. SDS was used as a surfactant. A homogenous stabilized oil-water emulsion was obtained. The oil-water separation test was performed using a simple gravity driven cell consisting of an open-ended beaker and a flask (Figure S1). A constant feed height was maintained throughout the course of the experiment such that a pressure of 0.1 bar is exerted due to the height of liquid column under gravity. Each separation test was carried out for at least five cycles with intermediate rinsing with water and ethanol to evaluate the membrane re-usability. The permeation flux (F) of the membrane was calculated using Equation (2), where ‘V’ is the total volume of fluid penetrating through the membrane, ‘A’ is the effective membrane area and ‘t’ is the separation test time.

(2) $$F=\frac{V}{A\times t}$$

After the separation experiment, permeates were evaluated for oil content using a total organic carbon analyzer (TOC) from TOC-VCHS, SHIMADZU, Kyoto, Japan and compared with the original oil content of the feed emulsion to determine the rejection percentage and membrane’s separation efficiency. The presence of oil droplets in the permeate was also examined by optical microscope.

## 3. Results and Discussion

**Figure 2:**
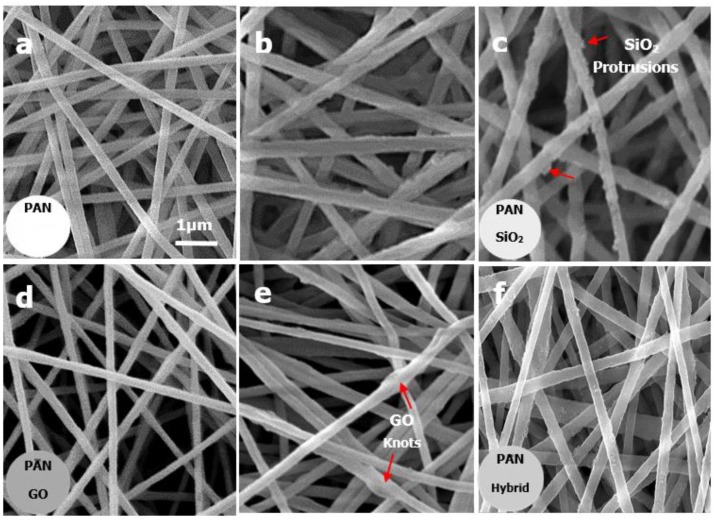
SEM images of developed membranes, (**a**) PAN, (**b**) PAN-4SiO_2_, (**c**) PAN-7.5SiO_2_, (**d**) PAN-0.5GO, (**e**) PAN-1.5GO, and (**f**) PAN-1.5GO-7.5SiO_2_. Inset shows digital images of the electrospun membranes (area = 20 cm^2^) used for separation test.

The appearance of the pristine PAN membrane was white while the appearance of membranes after adding inorganic fillers was observed to be changed after electrospinning. As seen in Figure 2 (inset) the whitish-colored pristine PAN based membrane became greyish with the addition of GO, which proves the uniform distribution of GO in a polymer solution. The addition of SiO_2_ did not produce any observable change in membrane appearance and it remained whitish in color due to the whitish color of silica particles. 

### 3.1. Morphological and Chemical Characterization of Developed Nanofiber Membranes

**Figure 3:**
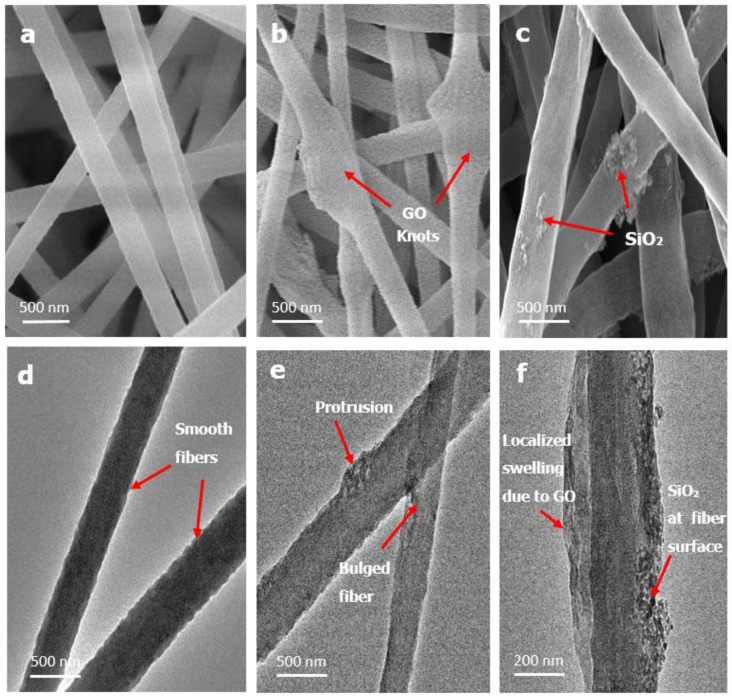
(**a**) SEM image of PAN nanofibers, (**b**) SEM image of fibers in PAN-GO membrane, (**c**) SEM image of fibers in PAN-SiO_2_ membrane, (**d**) TEM image of PAN nanofiber, (**e**) TEM image of fibers in hybrid membrane, (**f**) high magnification TEM image of fiber in hybrid showing nanofillers at bulged region.

Figure 2 shows the FESEM images of the pristine PAN, PAN-GO and PAN-SiO_2_ composite membranes, and PAN hybrid membrane. It is observed that the uniform/smooth PAN fibers were transformed to irregular/rough fibers with the addition of nanofillers. All the membranes were composed of entangled randomly-oriented fibers forming a highly porous, 3D non-woven structure. This 3D microporous structure offers low resistance to mass transfer and permits high liquid permeation through the membranes. The surface morphology of fibers was changed with the addition of silica nano-particles and submicron graphene oxide flakes. At low concentrations, most of the silica particles are embedded within PAN fibers. The SEM images show that with an increase in the concentration, silica particles tend to protrude at the fibrous surface, thus creating a rougher fiber surface. The formation of multi-level protrusions with increased SiO_2_ content can be attributed to varying degrees of evaporation of the solution jet, followed by rapid phase separation during the electrospinning process [27,28]. Moreover, it is observed that nanofiller addition create fibers with a thicker diameter, which could be due to the increase in the solution viscosity with increase in silica concentration. This higher viscosity of the electrospinning dope solution reduces the stretching of fibers during electrospinning and results in the formation of thicker fibers, which is also confirmed by other researchers [29]. We have also noticed that most of the graphene oxide is embedded within the PAN fibers, confirming high compatibility between PAN and GO. SEM images shown in Figure 2 also reveal the existence of localized fiber swelling or fiber knotting. The emergence of localized fiber swelling or knots with the GO addition could be due to the size mismatch between the sub-micron GO flakes and the PAN nanofibers. This can be viewed in Figure 3b. Similar fiber morphology was obtained in other studies where high interaction between PAN and GO caused the PAN molecules to adhere to the large GO sheets, forming swelled fibers or ellipsoids [30,31]. In the case of PAN hybrid fibers composed of PAN, GO, and SiO_2_, similar fiber morphologies were observed. The combination of nanoscale silica and micro-nano graphene oxide causes the appearance of nano protrusions and swelled fibers at some localized locations. The fibers were bulged due to the presence of silica and graphene oxide together, consequently increasing the fiber size. 

Transmission electron microscopy (TEM) of the hybrid membrane was done to examine the morphology of individual fibers. Figure 3a,d shows smooth nanofibers of a developed pristine PAN membrane. Figure 3e,f shows the low and high magnification TEM images of a hybrid PAN-GO-SiO_2_ membrane respectively. These TEM images depict non-uniform brightness at the swollen region compared to the smooth regions of the fiber. This appearance of dark and bright regions at the swollen or spindle-knotted region confirms the existence of GO sheets as a skeleton within the fibrous network, which may be due to the difference in electron density [32]. In addition, nano-level dark spots are also observed, which could be silica particles protruding at the surface. A highly magnified SEM image in Figure 3c reveals protrusions and agglomerations of SiO_2_ nanofillers. The reason for this agglomeration could be rapid evaporation of the DMF solvent during electrospinning, as mentioned earlier. 

**Figure 4:**
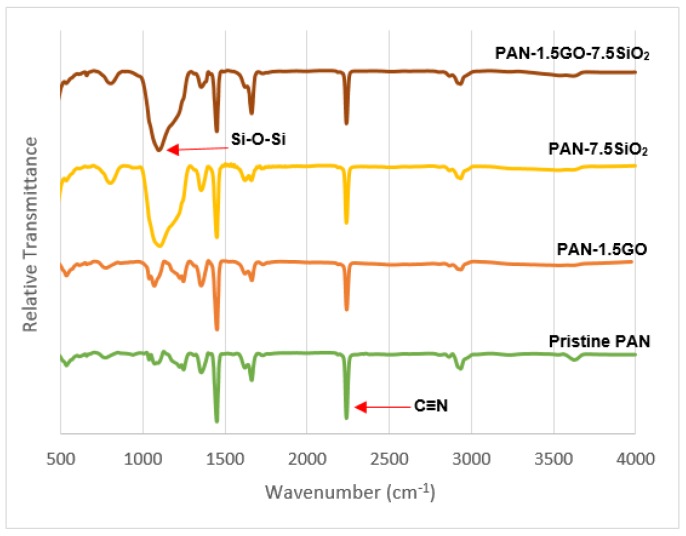
FTIR spectroscopy of developed membranes.

The chemical composition of developed membranes was investigated using FTIR spectroscopy. The FTIR spectra for pristine PAN and PAN with nanofillers is shown in Figure 4. All the spectra showed a characteristic peak of the nitrile group at 2242 cm^−1^ for PAN. While peaks from 2800–2950 correspond to C–H and –CH_2_– vibrations. The new peak appearing in membranes with SiO_2_ near 1100 cm^−1^ corresponds to Si–O–Si stretching [33,34]. When comparing the spectra of PAN-GO with that of pure PAN, it is observed that no new characteristic peaks appeared with the addition of GO, although the existence of GO could be confirmed by a small increase in peak intensity at 1072 cm^−1^ corresponding to C=O vibration of GO. The absence of new peaks in the PAN-GO composite was also observed in other studies, which could either be because there was no change in the chemical structure of PAN due to the addition of graphene oxide, or because GO might create bonds within the polymer chains, depicting good compatibility with PAN [35,36]. Thus, oxygen-containing groups in these composites would enhance the hydrophilicity as well as for the PAN-GO-SiO_2_ hybrid membrane.

### 3.2. General Characteristics of Developed Membranes 

Pore size and porosity of a membrane is very important, determining property for separation performance of membranes. Table 2 lists some of the important properties for the developed membranes such as fiber diameter, porosity %, membrane thickness, and average pore size. It was observed that all the membranes have pores ranging from 0.9–1.5 µm. There was no significant change in the pore size after addition of nanofillers. Thus, these membranes are expected to treat the emulsions that have droplet sizes of 1.5 µm or higher, and in the case of oil-in-water emulsions, it is usually the case. The developed membranes also show high porosity mainly due to the electrospinning process proving itself to be a method of producing highly porous membranes [37]. As mentioned in Table 2, the electrospun PAN based membranes have the average porosity of 85%. The highest porosity of 88% is obtained for the hybrid membrane composed of PAN with small additions of GO and SiO_2_. These high values of porosity with interconnected 3D structure and a pore size of around 1 µm is supposed to have better separation characteristics such as high permeability at low operating pressures. It can also be noted that the average fiber diameter of PAN increased with the addition of nanofillers. This increase in diameter is probably due to an increase in viscosity of the solution as nanofillers are dispersed in the PAN solution. 

### 3.3. Surface Wettability of Membranes

**Figure 5:**
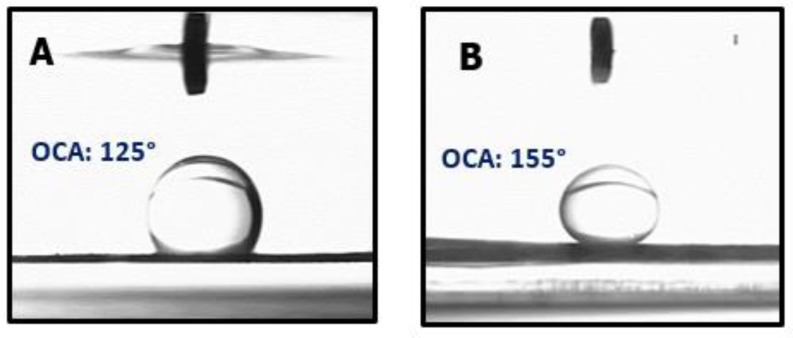
Underwater oil contact angle and oil droplet on (**A**) PAN and (**B**) PAN-1.5GO-7.5SiO_2_ nanofiber membranes.

Contact angle of water and oil-in-water was investigated for the developed membranes to evaluate their wetting behavior. The developed pristine PAN membrane was inherently hydrophilic, with a water contact angle of 15° and underwater oil-contact angle of 125° (Figure 5A). The underwater oil contact angle increased with the addition of inorganic nanofillers as shown in Table 3. The PAN membrane with a smaller GO addition of 0.5% shows an increment in oil contact angle up to 143°. When the content of GO further increased to 1.5%, the surface showed a 155° underwater oil contact angle combined with a water contact angle of 9°. All of the developed membranes were found to be hydrophilic mainly due to the hydrophilic PAN as membrane matrix. 

The surface characteristics of membranes in this study confirmed that the hierarchical structure due to the introduction of GO improves water affinity and superoleophobicity of the membrane as seen in other studies [31]. The GO knots as seen in Figure 3b are expected to be responsible for the aggregation and repulsion of oil droplets from the membrane surface. A similar trend was observed for membranes with addition of silica nano fillers. The reason for achieving high underwater contact angle in these membranes is that the surface hierarchical structure is composed of micro-nano features (silica protrusions), which cause the water to remain inside individual fibers, forming a triple-phase oil-water-solid condition; this reduces the contact area of oil and causes it to be rolled off from the surface (Video S1). Addition of silica alone and in combination with graphene oxide also revealed a superhydrophilic nature, which is due to the additional hydrophilic functionalities and similar underwater superoleophobic characteristics as depicted in Figure 5B.

### 3.4. Mechanical Testing

Mechanical properties of membranes are an important material aspect for any means of practical applications like reusability, handling, and anti-deformation capacity. Table 4 highlights the tensile behavior of the developed PAN membranes, including the composite and hybrid. The initial tensile strength of a pristine PAN electrospun membrane in this study was 6.4 MPa. Addition of nanofillers reduced the tensile strength of PAN composite membranes, except for 0.5% GO, mainly due to aggregation of the particles at higher concentrations, causing disturbance in the polymer chain interactions [34]. However, the ductility of composite has improved from 17% for pristine PAN to around 26% for the composites. This increase in ductility of the membrane can be due to an increase in fiber diameter after the addition of nanofillers. When graphene oxide was added in a small amount to the PAN solution, the strength increased from 6.4 MPa to 9.1 MPa at 0.5% GO loading, which could be because GO at low concentrations limits the chain disorientations of the polymer, thus improving strength and ductility [38,39,40]. Moreover, the strength of the hybrid membrane was increased by 21% and the membrane was stiffer as the elastic modulus had improved by 18%. This shows that the nanofillers in the hybrid membrane were uniformly embedded, resulting in a compact PAN nanofiber membrane. It has to be noted that the addition of nanofillers in concentrations more than 7.5% SiO_2_ or 1.5% GO proved detrimental to the membrane strength and handling, and thus the composition of the hybrid was limited to these concentrations. Moreover, at higher nanofiller concentrations, electrospinning at preset parameters became difficult due to an increase in viscosity of the solution and tendency of particle agglomeration during electrospinning. 

### 3.5. Performance Evaluation of Membranes in Oil Water Separation Test

**Figure 6:**
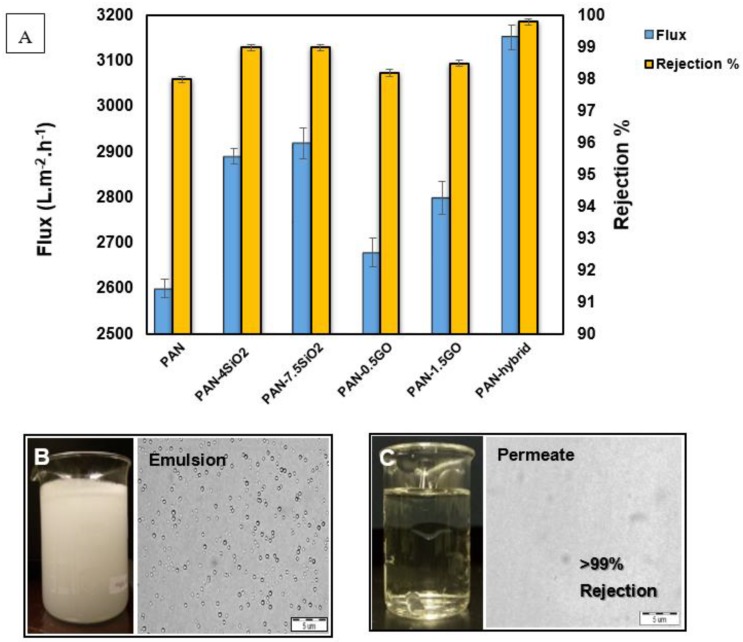
(**A**) Flux of permeate and rejection% of oil for the developed membranes with different nanofillers; (**B**) optical image of milky white emulsion with oil droplets; (**C**) optical image of permeate from hybrid membrane showing no oil droplets.

The developed electrospun membranes consisting of pristine PAN, PAN with GO, PAN with SiO_2,_ and hybrid PAN-GO-SiO_2_ membrane were tested by fixing them on a dead-end filtration cell. The oil water emulsion was passed through the cell under a pressure of 0.1 bar (height of liquid column under gravity). For all the developed membranes, the separation test was performed under gravity without applying any external pressure. After separation tests, permeates were evaluated for oil content using a total organic carbon analyzer and compared with the original oil content of the feed emulsion to determine the rejection percentage and membrane’s separation efficiency. Additionally, the presence of oil droplets in the permeate was also examined by optical microscope. Figure 6A shows the water flux and rejection percentage of oil after the separation test for the developed membranes. The pristine PAN membrane in this study showed an appreciable water flux of 2600 L m^−2^ h^−1^. Flux was increased with increased concentration of SiO_2_ while the increase in flux with GO was only marginal as compared to pristine PAN, which may be due to low concentration of GO in the membrane. The PAN membrane, with a highly porous entangled structure, showed a separation efficiency of 98%, while adding hydrophilic silica and antifouling graphene oxide further enhances the rejection percentage of oil close to 99%, which may be due to surface features of the fibers. Another reason for this high rejection rate of oil in all the developed membranes is the pore size achieved from the electrospinning process. The highly porous structure of nanofiber membranes with hydrophilic characteristics of PAN and nanofillers helped in achieving an appreciable flux rate even under a gravity separation test. The initial feed emulsion, which was milky (Figure 6B), turns transparent after passing through the fabricated membranes, proving high separation efficiency. In the case of the PAN-1.5GO-7.5SiO_2_ hybrid membrane, an average flux of 3151 L m^−2^ h^−1^ was achieved with a separation efficiency of more than 99% (Figure 6C). This improvement in flux in the hybrid membrane can be attributed to the change in morphology of fibers and more hydrophilic groups in the polymer chain after adding silica and graphene oxide. 

**Figure 7:**
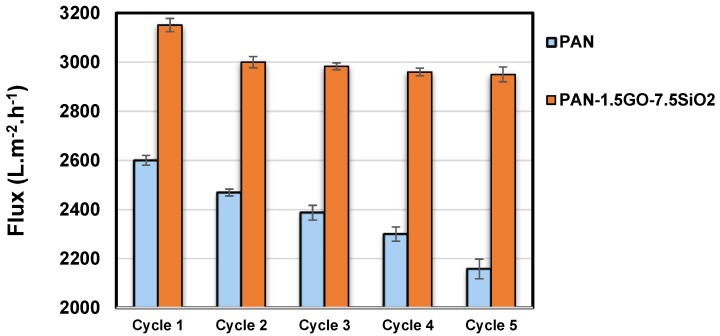
Flux for the permeate of pristine PAN membrane and PAN-1.5GO-7.5SiO_2_ hybrid membrane for 5 consecutive cycles.

To evaluate the reusability and antifouling nature of developed membranes, the oil-water separation test for five cycles was performed. As depicted in Figure 7, the highest flux of 3151 L m^−2^ h^−1^ was achieved in the first cycle of separation for the hybrid membrane. The purpose of adding GO with SiO_2_ in the hybrid was to enhance the hydrophilicity together with the added antifouling characteristics. The antifouling behavior from graphene oxide can be seen in the separation test for five repeated cycles in the case of the hybrid membrane. This finding was in accordance with the previous study of Zhang et al. [30], where in the spindle-knots formed with the incorporation of GO, a better fouling resistance resulted. In the case of the pristine PAN membrane, a gradual decrease in flux was observed, which might be due to the fouling of fibers under continuous oil-water treatment. The rejection % of oil from the hybrid membrane remained constant in all of the separation cycles. It has to be noted that the small decline in flux after the first test (cycle 1) was due to an inability of the cleaning operation to get rid of any tiny oil particles in the membrane pores. The flux remains about the same later on after cycle 2, which depicts an antifouling nature of this membrane. Therefore, a proper cleaning technique has to be investigated for cleaning membranes for flux recovery. The hybrid membrane was easy to handle and can be used multiple times for the oil water separation test. 

## 4. Conclusions

PAN hybrid membranes with silica and graphene oxide fillers were successfully fabricated using a one-step electrospinning process. The incorporation of silica nanoparticles introduced micro-nano protrusions on the surface of electrospun nanofibers, while the incorporation of graphene oxide resulted in the formation of knots within the smooth fibrous network of PAN, and the frequency of these features increased with an increase in filler content. Adding GO and SiO_2_ in combination to develop hybrid membrane resulted in the improvement of membrane performance due to the hierarchical structure of fibers and additional hydrophilic groups. The hybrid membrane was able to separate water from oil-water emulsion more efficiently than other developed membranes even after five cycles of separation, displaying its antifouling behavior and reusability.

## Figures and Tables

**Table 1:** Electrospinning compositions of composite and hybrid membrane.

Sample Label	Composition
PAN	Pristine PAN
PAN-4SiO_2_	PAN + 4 wt % SiO_2_
PAN-7.5SiO_2_	PAN + 7.5 wt % SiO_2_
PAN-0.5GO	PAN + 0.5 wt % GO
PAN-1.5GO	PAN + 1.5 wt % GO
PAN-1.5GO-7.5SiO_2_	PAN + 1.5 wt % GO + 7.5 wt % SiO_2_

**Table 2:** Characteristics of the developed membranes.

Membrane	Fiber Diameter (nm)	Porosity (%)	Membrane Thickness (μm)	Average Pore Size (μm)
PAN	220	85	100	1.5
PAN-4SiO_2_	300	87	105	1
PAN-7.5SiO_2_	310	85	107	1.05
PAN-0.5GO	307	85	96	0.95
PAN-1.5GO	290	86	84	1.2
PAN-1.5GO-7.5SiO_2_	320	88	110	1.3

**Table 3:** Water contact angle (WCA) and underwater oil contact angle (OCA) for developed membranes.

Membrane	PAN	PAN-4SiO_2_	PAN-7.5SiO_2_	PAN-0.5GO	PAN-1.5GO	PAN-1.5GO-7.5SiO_2_
WCA	15°	12°	10°	10°	9°	7°
OCA	125°	145°	150°	143°	155°	155°

**Table 4:** Mechanical properties of developed membranes with different nanofillers.

Membrane	Tensile Strength (MPa)	Elongation (%)	Elastic Modulus (MPa)
PAN	6.4 ± 0.09	17 ± 0.8	108 ± 1.5
PAN-4SiO_2_	4.9 ± 0.04	26 ± 1.4	70 ± 5.9
PAN-7.5SiO_2_	4.6 ± 0.14	25.1 ± 1.3	77 ± 4.6
PAN-0.5GO	9.1 ± 0.11	25.7 ± 3.2	98 ± 0.5
PAN-1.5GO	4.8 ± 0.02	26.9 ± 1	65 ± 0.9
PAN-1.5GO-7.5SiO_2_	7.8 ± 0.3	13.9 ± 1.5	128 ± 1.8

## Supplementary Materials

The following are available online at http://www.mdpi.com/1996-1944/12/2/212/s1, Figure S1: Flow cell used in the oil-water separation experiments, Video S1: video depicting oil droplet movement over PAN-1.5GO-7.5SiO_2_ hybrid membrane surface.
